# Editorial: Microbiome in IBD: From Composition to Therapy, Volume II

**DOI:** 10.3389/fphar.2023.1184269

**Published:** 2023-04-05

**Authors:** Ning-Ning Liu, Peijian He, Zhanju Liu, Ruixin Zhu, Yinglei Miao, Chenggong Yu, Lixin Zhu

**Affiliations:** ^1^ State Key Laboratory of Oncogenes and Related Genes, Center for Single-Cell Omics, School of Public Health, Shanghai Jiao Tong University School of Medicine, Shanghai, China; ^2^ Division of Digestive Diseases, Department of Medicine, Emory University School of Medicine, Atlanta, GA, United States; ^3^ Center for IBD Research, Department of Gastroenterology, The Shanghai 10th People’s Hospital, Tongji University, Shanghai, China; ^4^ Department of Gastroenterology, The Shanghai 10th People’s Hospital, Department of Bioinformatics, School of Life Sciences and Technology, Tongji University, Shanghai, China; ^5^ Department of Gastroenterology, First Affiliated Hospital of Kunming Medical University, Yunnan Institute of Digestive Diseases, Kunming, China; ^6^ Department of Gastroenterology, Nanjing Drum Tower Hospital, The Affiliated Hospital of Nanjing University Medical School, Nanjing, China; ^7^ Guangdong Institute of Gastroenterology, Guangdong Provincial Key Laboratory of Colorectal and Pelvic Floor Diseases, Department of Colorectal Surgery, The Sixth Affiliated Hospital, Sun Yat-sen University, Guangzhou, China

**Keywords:** inflammatory bowel disease, microbiome, ulcerative colitis, Crohn’s disease, interaction

Inflammatory bowel disease (IBD) is a chronic, non-specific, immune-mediated, inflammatory intestinal disease, which is divided into ulcerative colitis (UC) and Crohn’s disease (CD). IBD debilitates human physical and psychosocial strength and has a rapidly increasing incidence rate in newly industrialized countries (Ng et al., 2017). Although the etiology of IBD remains unclear, emerging sources of evidence have suggested that gut microbiome may significantly affect the pathogenesis, progression, and prognosis of IBD both in animal models and humans directly and/or indirectly (Ni et al., 2017). Therefore, this Research Topic aims to discuss the alteration of the gut microbiome in IBD and its relationship with the treatment of IBD.

Extensive studies have explored the value of multidimensional information, including microbiome data, especially based on SCFA-producing bacteria or certain intestinal microbes, clinical data, and serologic markers, in predicting the prognosis of IBD, which provided higher accuracy and sensitivity. The composition and function of the gut microbiome and their metabolic levels in IBD are reported to be significantly associated with disease severity and response to biological therapy. Furthermore, comprehensive multi-omics data are now integrated to build predictive models in IBD, including metagenomics, proteomics, and metabolomics. Notably, Liu et al. reported that proteomic analysis can identify drug targets on the basis of intestinal biopsy tissue first and serve as predictive biomarkers in response to infliximab, a rescue therapy used for treating refractory UC. In addition to predicting the pathological state of IBD, assessing the activity of IBD is also important, especially in pediatric CD. Li et al. utilized Spearman’s rank correlation test and kappa consistency analysis in pairwise comparisons of several indexes, including MaRIA, CECDAI, PCDAI, and SES-CD. It was demonstrated that MGCE and MRE were beneficial in evaluating small bowel lesions in pediatric CD, being both non-invasive and highly consistent with colonoscopy.

As the first-line therapy for IBD, anti-TNF-α monoclonal antibodies, such as infliximab and adalimumab, could reduce the severity of disease and improve the diversity and composition of gut microbiota in patients with IBD. However, approximately 70% of patients with IBD exhibit primary non-response or secondary loss of response when receiving biological therapy. To tackle this problem, Xiao et al. showed that *Bifidobacterium longum* CECT 7894 improved the efficacy of infliximab for DSS-induced colitis *via* the regulation of the intestinal microbiota composition and structure through 16S rRNA sequencing and bile acid metabolism process analyzed by targeted metabolomics. However, the mechanism behind how anti-TNF-α monoclonal antibodies regulate gut microbiota is still not comprehensively illustrated. Chen et al. indicated that distinct alterations of gut microbiota were related to the efficacy of adalimumab in CD patients. Although they discovered that the efficacy of adalimumab was not associated with the location of disease lesions, a retrospective cohort study recently indicated that lesion location can alter their prognosis. For example, CD patients with the L4-esophagogastroduodenal (EGD) phenotype are associated with a better prognosis and lower morbidity of complications (Weng et al.).

Fecal microbiota transplantation (FMT), an emerging high-efficiency treatment, was proposed to improve the efficacy and reduce the side effects of traditional therapy. Several studies have demonstrated that FMT improves the gut microbiome composition and diversity of patients with IBD. Pu et al. reviewed the therapeutic mechanisms of FMT combined with biologics in IBD. Moreover, the authors also pointed out the challenges and potential risks of FMT when applied to adjuvant therapy.

Additionally, Rao et al. discovered that Shenling Baizhu powder (SBP), a type of traditional Chinese medicine, alleviates colitis in rats treated with TNBS. This study revealed that SBP could increase the secretion of mucin and tight junction and inhibit apoptosis to improve intestinal epithelial permeability. It was suggested that there exist certain components in traditional Chinese medicine which may influence the intestinal microecology in IBD and provide new pharmaceutical therapeutic perspectives.

Finally, the state-of-the-art causal inference analysis proved that *IFNG* and *GBP5* were IBD subtype-regulators that triggered more intense innate immunity and inflammatory responses in CD than those in UC (Gao et al.).

Taken together, the studies included in this themed Research Topic have provided novel microbial and therapeutic insights into IBD treatment. With the continuous development of sequencing technologies and bioinformatics methods, an accurate and personalized understanding of the pathogenesis and progression of IBD can be anticipated.
